# Supplementary material for: The Effect of Cation Disorder on Ferroelectric Properties of SrxBa1−xNb2O6 Tungsten Bronzes
Source: Materials (Basel). 2019 Apr 10;12(7):1156. doi: 10.3390/ma12071156 (PMC6480143; doi:10.3390/ma12071156)
Supplement: Supplementary file 1 [file materials-12-01156-s001.pdf]

materials

Supplementary Information: The Effect of Cation Disorder on Ferroelectric Properties of $Sr_xBa_{1-x}Nb_2O_6$ (SBN) Tungsten Bronzes

Solveig S. Aamlid, Sverre M. Selbach and Tor Grande *

Department of Materials Science and Engineering, NTNU Norwegian University of Science and Technology, NO-7491 Trondheim, Norway; solveig.s.aamlid@ntnu.no (S.S.A.), selbach@ntnu.no (S.M.S.)

* Correspondence: grande@ntnu.no

Received: 28 February 2019; Accepted: 8 April 2019; Published: date

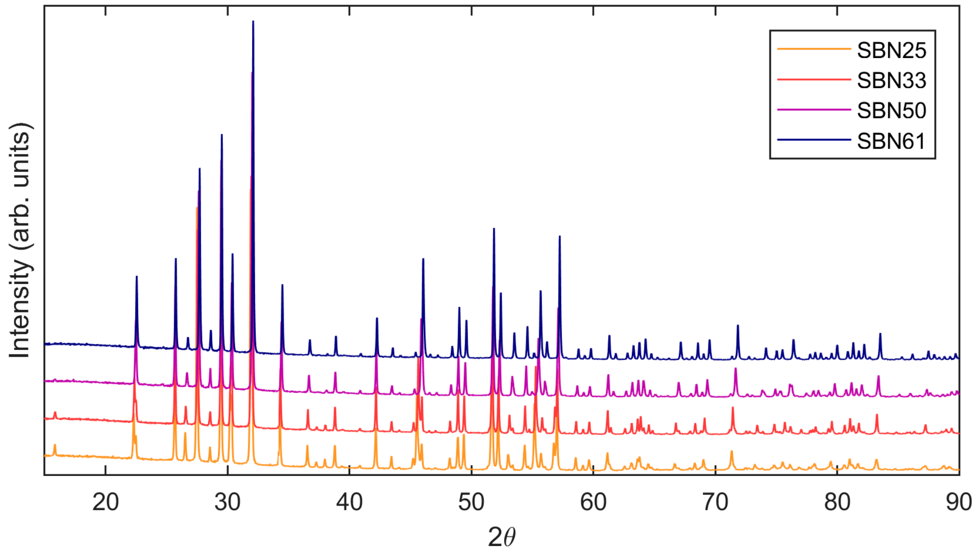

Figure S1. Diffractograms of the four SBN compositions heat treated at 800 °C.

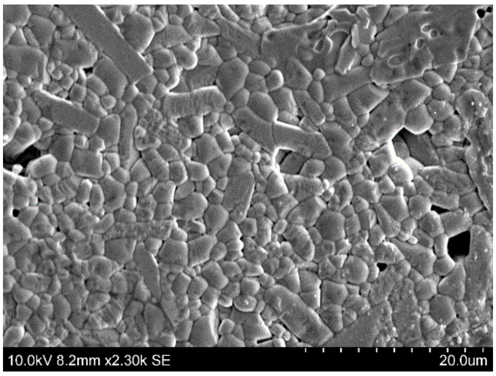

(**a**)

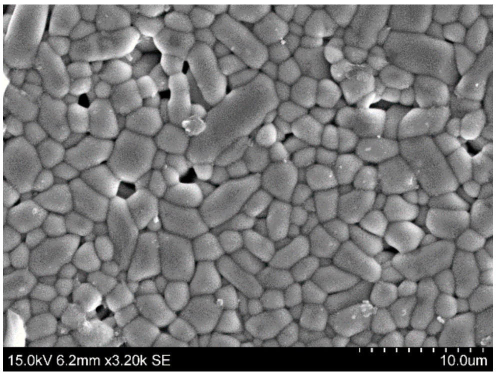

(**b**)

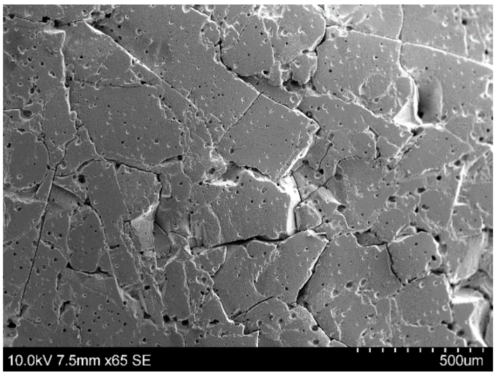

(**c**)

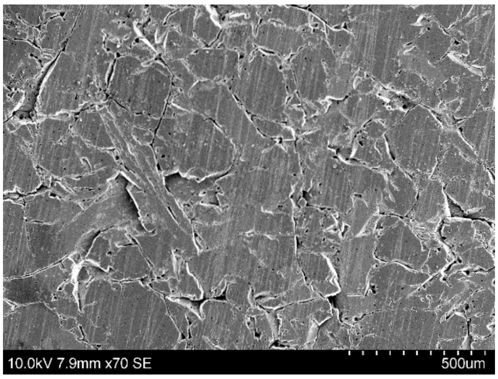

(**d**)

Figure S2. SEM micrographs of the four samples (**a**) SBN25, (**b**) SBN33, (**c**) SBN50, and (**d**) SBN61. Note the difference in the scale bars showing that the SBN50 and SBN61 are significantly coarser due to their higher sintering temperature (1400 °C).

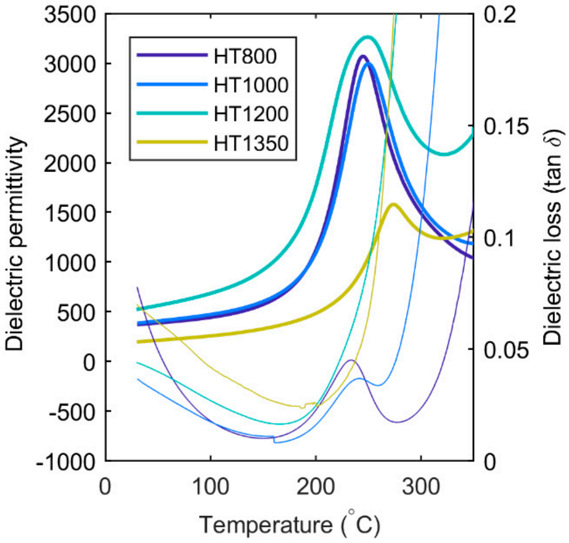

(**a**)

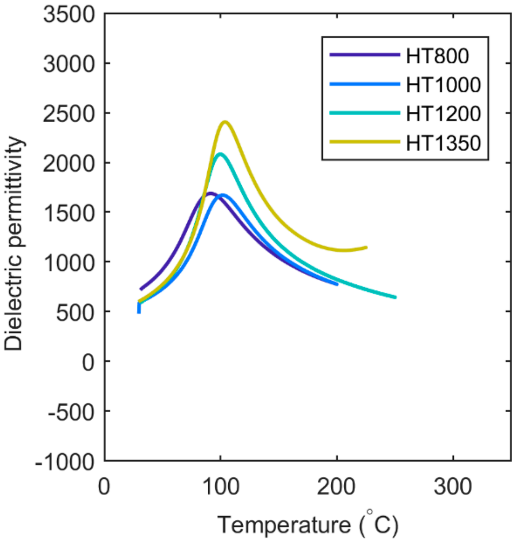

(**b**)

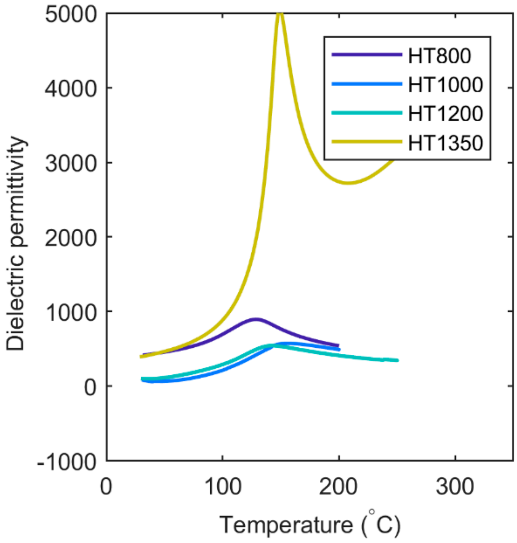

(**c**)

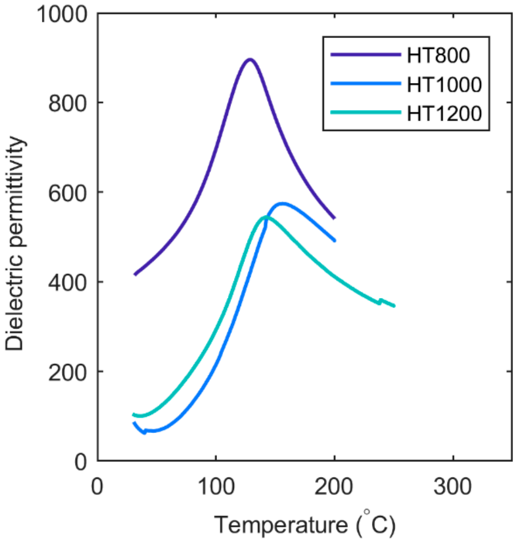

(**d**)

Figure S3. Dielectric permittivity and loss for (**a**) SBN25, and dielectric permittivity for (**b**) SBN61 at 1000 Hz, and dielectric permittivity for (**c**,**d**) SBN50 with two different y-axes. Loss curves are not

shown for SBN50 and SBN61 due to noisy measurements for the large grain sizes. The absolute value for the dielectric permittivity of SBN25 HT1350 is low because it is the fifth cycle of measurements up to 400 °C, which is enough to reoxidize the sample.

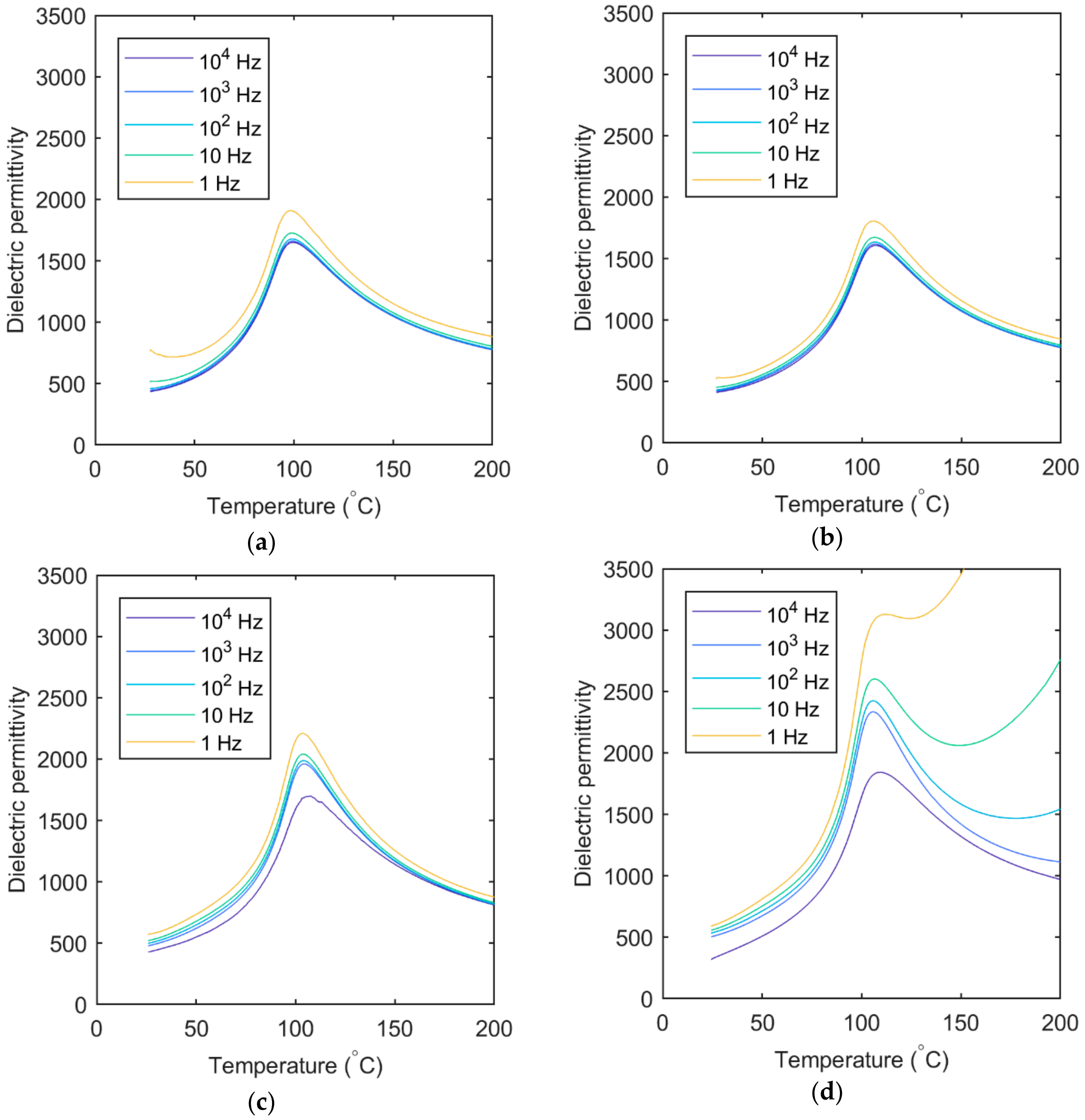

Figure S4. Dielectric permittivity at 1–10⁴ Hz shown for SBN61 heat treated at (**a**) 800 °C; (**b**) 1000 °C; (**c**) 1200 °C; and (**d**) 1350 °C. The dispersion in T_C is small for all the samples, and not affected by heat treatments; the permittivity decrease with the frequency; and the free charge carriers in the sample heat treated at 1200 °C and 1350 °C cause the absolute value of the apparent permittivity to increase.

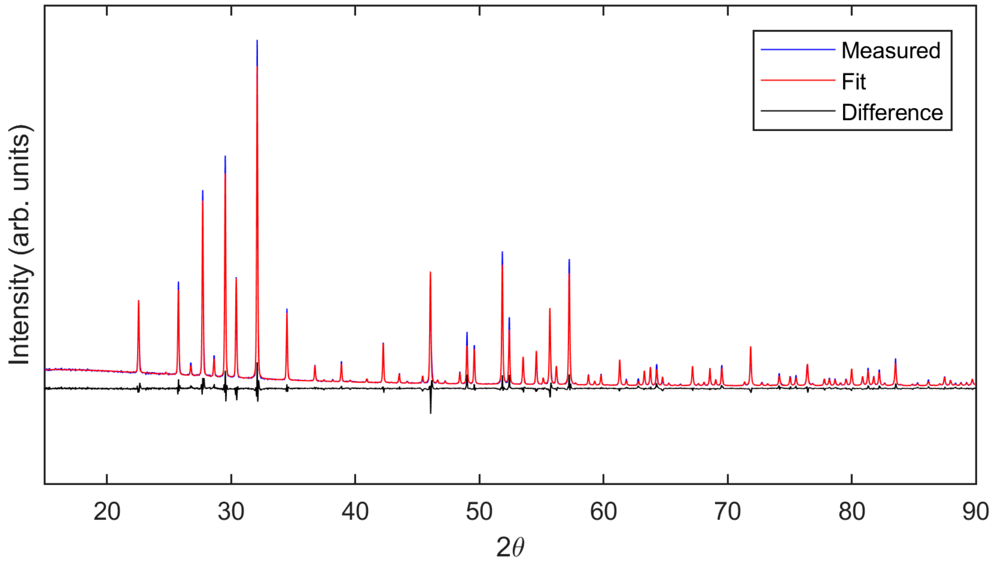

Figure S5. A typical fit and comparison with the measured diffractogram for the Rietveld refinements (SBN61 heat treated at 800 °C).

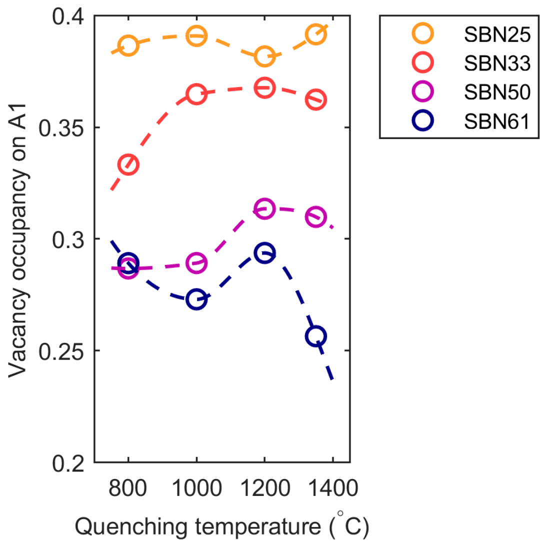

Figure S6. Vacancy distribution on the A1 site for all the compositions and quenching temperatures.

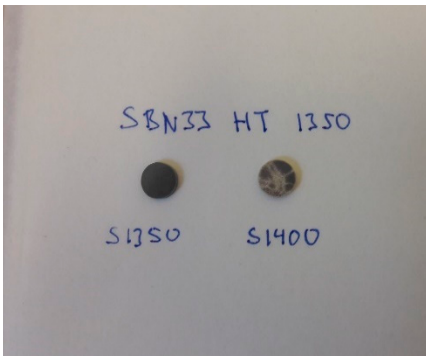

Figure S7. SBN33 pellets sintered at 1350 °C (left) and 1400 °C (right) after quenching from 1350 °C. The finer microstructure and lack of cracking in the sample that is sintered at a lower temperature, makes the sample more grey, which is a sign of oxygen vacancies/reduced niobium.

Table S1. Some extracted values from the from the synchrotron data, for the four compositions heat treated at 800 °C.

Sample	Rwp	a	a-error	c	c-error	SrA1	SrA2
SBN25 HT800	3.96617	12.48254	0.00017	3.97682	0.00006	0.62275	0.00112
SBN33 HT800	4.06528	12.48049	0.00015	3.97039	0.00005	0.65085	0.08707
SBN50 HT800	2.94989	12.47399	0.00017	3.94657	0.00007	0.72321	0.26339
SBN61 HT800	2.52359	12.456	0.00015	3.93517	0.00006	0.7234	0.4008

Table S2. The refined values for the samples heat treated at 800 °C for (**a**) SBN25, (**b**) SBN33, (**c**) SBN50, and (**d**) SBN61

(**a**)

Site	Mp	Atom	x	y	z	Occupancy	Biso
Nb1	2	Nb+5	0	0.5	-0.017652	1	1.77(2)
Nb2	8	Nb+5	0.07391(10)	0.21049(8)	-0.038(2)	1	1.77(2)
A1	2	Sr+2	0	0	0.445(3)	0.623(6)	1.77(12)
		Ba+2	0	0	0.445(3)	0	1.77(12)
A2	4	Sr+2	0.17217(9)	0.67217(9)	0.473(2)	0.001(3)	2.78(3)
		Ba+2	0.17217(9)	0.67217(9)	0.473(2)	0.9375	2.78(3)
O1	8	O-2	0.3406(8)	0.0068(5)	-0.094(3)	1	1.9026
O2	8	O-2	0.1409(6)	0.0710(7)	-0.100(3)	1	1.934
O3	4	O-2	0.2856(5)	0.7856(5)	-0.093(4)	1	0.134
O4	2	O-2	0	0.5	0.414(8)	1	3.8448
O5	8	O-2	0.2933(6)	0.4274(7)	0.419(4)	1	2.8816

(**b**)

Site	Mp	Atom	x	y	z	Occupancy	Biso
Nb1	2	Nb+5	0	0.5	-0.017652	1	1.59141
Nb2	8	Nb+5	0.07445(10)	0.21076(9)	-0.04776	1	1.59141
A1	2	Sr+2	0	0	0.47272	0.651(4)	1.88258
		Ba+2	0	0	0.47272	0	1.88258
A2	4	Sr+2	0.17222(10)	0.67222(10)	0.46022	0.087(2)	2.85155
		Ba+2	0.17222(10)	0.67222(10)	0.46022	0.8375	2.85155
O1	8	O-2	0.34209	0.00934	-0.11177	1	1.9026
O2	8	O-2	0.14345	0.06923	-0.11565	1	1.934
O3	4	O-2	0.28449	0.78449	-0.10371	1	0.134
O4	2	O-2	0	0.5	0.40659	1	3.8448
O5	8	O-2	0.29396	0.42479	0.3913	1	2.8816

(**c**)

Site	Mp	Atom	x	y	z	Occupancy	Biso
Nb1	2	Nb+5	0	0.5	-0.017652	1	1.67(2)
Nb2	8	Nb+5	0.07458(11)	0.21150(10)	-0.009(3)	1	1.67(2)
A1	2	Sr+2	0	0	0.487(5)	0.723(8)	1.92(11)
		Ba+2	0	0	0.487(5)	0	1.92(11)
A2	4	Sr+2	0.17202(12)	0.67202(12)	0.506(3)	0.263(4)	2.96(5)
		Ba+2	0.17202(12)	0.67202(12)	0.506(3)	0.625	2.96(5)
O1	8	O-2	0.3377(10)	0.0066(7)	-0.085(4)	1	1.9026

O2	8	O-2	0.1359(7)	0.0708(9)	-0.061(5)	1	1.934
O3	4	O-2	0.2871(7)	0.7871(7)	-0.083(4)	1	0.134
O4	2	O-2	0	0.5	0.406(8)	1	3.8448
O5	8	O-2	0.2936(8)	0.4277(10)	0.445(5)	1	2.8816

(**d**)

Site	Mp	Atom	x	y	z	Occupancy	Biso
Nb1	2	Nb+5	0	0.5	-0.017652	1	1.40(2)
Nb2	8	Nb+5	0.07494(12)	0.21158(11)	0.003(2)	1	1.40(2)
A1	2	Sr+2	0	0	0.508(4)	0.723(9)	1.63(12)
		Ba+2	0	0	0.508(4)	0	1.63(12)
A2	4	Sr+2	0.17209(14)	0.67209(14)	0.515(3)	0.401(4)	2.75(6)
		Ba+2	0.17209(14)	0.67209(14)	0.515(3)	0.4875	2.75(6)
O1	8	O-2	0.3368(11)	0.0077(8)	-0.097(3)	1	1.9026
O2	8	O-2	0.1375(8)	0.0687(10)	-0.057(5)	1	1.934
O3	4	O-2	0.2867(7)	0.7867(7)	-0.063(5)	1	0.134
O4	2	O-2	0	0.5	0.408(8)	1	3.8448
O5	8	O-2	0.2943(9)	0.4269(11)	0.455(4)	1	2.8816

Table S3. Rietveld refinement details for the VCT Refinements: The cation coordinates are allowed to change as constrained by symmetry, the oxygen z-coordinates and all thermal parameters are set equal to the output from the synchrotron refinement, and the strontium/vacancy occupancy of A1/A2 is allowed to change as constrained by composition. A TCHZ peak type, simple axial model, and sixth order Chebychev polynomial was used to model the instrument and background. A table of the refined values for the weighted average error, a and c lattice parameter with absolute errors, and strontium/vacancy distribution on the A2 site.

Sample	Rwp	a	a-error	c	c-error	SrA1	VA1
SBN25 HT800	7.27247	12.48913	0.00016	3.98027	0.00006	0.61351	0.77298
SBN25 HT1000	7.2688	12.48535	0.00016	3.98152	0.00006	0.60918	0.78164
SBN25 HT1200	9.00961	12.48606	0.00022	3.981	0.00008	0.61834	0.76332
SBN25 HT1350	8.87813	12.48725	0.00022	3.98137	0.00008	0.60847	0.78306
SBN33 HT800	8.00817	12.4866	0.00012	3.97223	0.00004	0.66674	0.66652
SBN33 HT1000	7.76612	12.48247	0.00011	3.97233	0.00004	0.63524	0.72952
SBN33 HT1200	7.9074	12.48305	0.00011	3.97329	0.00004	0.63237	0.73526
SBN33 HT1350	7.02209	12.48267	0.00009	3.97303	0.00003	0.63767	0.72466
SBN50 HT800	7.71451	12.47495	0.00011	3.95042	0.00004	0.71319	0.57362
SBN50 HT1000	7.63008	12.47117	0.00011	3.95077	0.00004	0.71077	0.57846
SBN50 HT1200	8.1666	12.47091	0.00012	3.94994	0.00005	0.68649	0.62702
SBN50 HT1350	8.4624	12.46928	0.00012	3.95076	0.00005	0.69013	0.61974
SBN61 HT800	7.67679	12.45685	0.00019	3.93646	0.00006	0.71081	0.57838
SBN61 HT1000	6.64755	12.4537	0.00007	3.93731	0.00003	0.72699	0.54602
SBN61 HT1200	6.9101	12.45406	0.00007	3.9374	0.00003	0.70626	0.58748
SBN61 HT1350	7.52062	12.45345	0.00008	3.93758	0.00003	0.7435	0.513

© 2019 by the authors. Submitted for possible open access publication under the terms and conditions of the Creative Commons Attribution (CC BY) license (http://creativecommons.org/licenses/by/4.0/).
